# Aripiprazole Provoking Gambling Disorder in a Patient With Opioid Use Disorder: A Case Report

**DOI:** 10.7759/cureus.71012

**Published:** 2024-10-07

**Authors:** Lauren J Woyak, Waqas Yasin

**Affiliations:** 1 Psychiatry, Medical College of Wisconsin, Wausau, USA; 2 Psychiatry and Behavioral Medicine, Medical College of Wisconsin, Wausau, USA

**Keywords:** abilify, aripiprazole, compulsive gambling, depression, impulsivity, opioid use disorders, suicide

## Abstract

Aripiprazole is an atypical antipsychotic used to treat certain mood disorders, such as bipolar disorder or schizophrenia, or as an adjunct therapy for treatment-resistant depression. In 2016, the FDA reported compulsive behaviors or uncontrollable urges can be associated with aripiprazole use. We report a 37-year-old Caucasian female with a past medical history of unspecified depressive disorder and opioid use disorder, who was treated with aripiprazole (Abilify) and then experienced compulsive gambling resulting in a suicide attempt. The patient reported playing scratch-off lottery tickets since childhood; however, she reported an ability to gamble responsibly and infrequently until one month before the admission. The patient started aripiprazole 10 mg daily, as an adjunct therapy for unspecified depressive disorder, approximately one year before the admission. We discuss the possibility of aripiprazole use, in combination with previous poor impulse control, intensifying recreational gambling to a level of gambling disorder. Because of the severity of these side effects, prior to beginning this medication, prescribing practitioners should consider if their patients have a history of compulsive behaviors and inform them of the potential for decreased impulse control with its use.

## Introduction

Aripiprazole (Abilify) is an antipsychotic medication that can be used in combination with other antidepressants to treat resistant depression. Aripiprazole works on both the dopamine and serotonin systems in the brain, which provides an added effect in combination with other psychotropic medications [1]. However, a rare, yet serious, side effect includes compulsive gambling [1-3]. Gambling disorder is a diagnosable psychiatric condition defined by the Diagnostic and Statistical Manual of Mental Disorders-Fifth Edition (DSM-5), which describes it as "Persistent and recurrent problematic gambling behavior leading to clinically significant impairment or distress" with four additional qualifying factors [4]. While rare, it is well worth mentioning to future prescribers and consumers of aripiprazole as multiple plaintiffs have filed and won lawsuits against Abilify, stating they began excessively gambling, eating, shopping, or having sex after starting Abilify [1]. In 2016, the FDA distributed a warning stating aripiprazole could cause the above side effects, and most cases of compulsive gambling will occur within a few days to one year after starting the medication [2,5]. These side effects can lead to life-altering circumstances, as reported in the following case, and it appears patients with previously reported impulsive behaviors and other confounding factors are at a greater risk for experiencing these dangerous side effects. For those who develop gambling disorder after beginning aripiprazole, there is typically an improvement in symptoms shortly after discontinuing the medication [6]. In this study, we report a case of a 37-year-old female with a history of opioid use disorder who developed a gambling disorder less than a year after beginning aripiprazole.

## Case presentation

A 37-year-old Caucasian, married, employed female with a history of unspecified depressive disorder and opioid use disorder voluntarily admitted herself to the inpatient psychiatric unit after attempting suicide by overdose; the patient ingested 44 gabapentin and 10 cyclobenzaprine which belonged to her mother. For the past month, she had been gambling large amounts of money, in the thousands of dollars, and felt her guilt was too great to handle if she lost another paycheck. Despite this, she could not stop her urge to gamble and her desire to win. Because of this, she indicated her intention was to gamble to win and make up for her losses or to commit suicide if she lost. After the patient lost, she attempted suicide, sent a suicide text to her spouse, and then lay in her son's room, prepared not to wake up. Remarkably, the patient awoke the next morning when her spouse found her. She then called her psychiatrist, who suggested that she admit herself to the inpatient psychiatric unit. The patient was medically stabilized in the ER and then cleared to be admitted voluntarily to the unit. Because the patient has a history of opioid use disorder, the patient was assessed for withdrawal symptoms, and liver function tests were completed and resulted within normal limits (Table 1).

**Table 1:** Clinical data include liver function test values from the patient, along with the corresponding reference ranges. AST: aspartate transaminase; ALT: alanine transaminase

Liver function tests	Patient value	Reference range
AST (U/L)	23	0-35
ALT (U/L)	30	0-45

**Figure 1:**
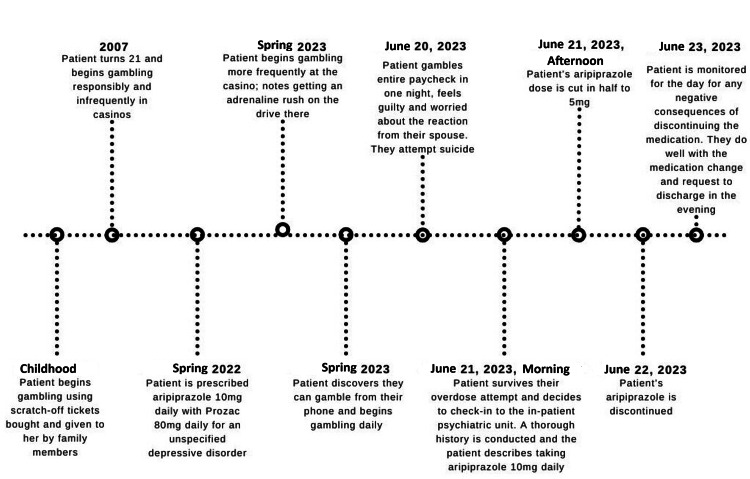
Chronopathogram of the patient's events from childhood to admission.

On intake, the patient indicated her gambling began with scratch-off tickets as a kid, which then became buying lottery tickets as a young adult, and then going to the casino when she was 21 years old. She indicated that she was always able to gamble within her means, gambling infrequently, and only spending a certain amount of money when she did gamble. This was true until the spring of 2023 when she began visiting the casino more frequently and subsequently discovered online gambling. It should be mentioned that the patient's husband also attested to this statement and noted an increase in gambling within the past year. While interviewing the patient, it was discovered she began aripiprazole 10 mg daily for treatment-resistant depression about one year before the admission, and her gambling habits increased within this same time frame (Figure 1). 

The patient has been married for 15 years, has two kids, and has a bachelor’s degree in accounting. As an adolescent, the patient was diagnosed with an unspecified depressive disorder, later with social anxiety, and subsequently with shift work disorder as an adult. The patient was prescribed Prozac, lorazepam, and modafinil, respectively. The patient had a significant maternal family history of mental health disorders, including depression and a second-degree relative with a suicide attempt.

The patient was awake and interactive during the interview and oriented to person, place, time, and circumstances. Her thought content was logical and devoid of any delusions or phobias, and the patient denied any acute suicidal/homicidal ideations, plans, or intent at the time of the interview. Her memory was grossly intact as she was able to describe recent and past experiences in detail; however, it was not formally tested as she showed no concerns for memory impairment. Her intelligence was estimated to be average.

Because aripiprazole is a drug with an FDA warning for new impulse control problems associated with its use, it was tapered to 5 mg for one day and then discontinued the next day (Figure 1). Because liver function tests were normal and the patient did not show any signs of opioid withdrawal, naltrexone 25 mg PO was started for opioid use disorder, indications of impulsivity, and patient interest in starting the medication. The patient was prescribed modafinil PRN for shift work disorder for the past five years; however, this was held for the course of the hospitalization as the patient was not working while hospitalized, was able to maintain an adequate sleep schedule, and was monitored closely for signs or symptoms of withdrawal. Lorazepam was also held as the patient was adequately able to manage her anxiety symptoms by using coping techniques and hydroxyzine 25 mg PO as needed. 

Over the course of the hospitalization, the patient showed improvements in mood, enhanced her involvement/interaction with others on the unit, despite her self-reported social anxiety, and remained consistent in her denial of suicidal ideation or homicidal ideation. She became futuristic in her thinking, talking about plans to see her kids and husband, and even showed humor in the appropriate moments. The patient indicated no side effects to the medication changes, did not show any concerns for acute danger, and indicated she was not thinking about gambling at the time of the interview. After a review of the patient’s history, symptoms upon admission, mental status examination, collateral information from her spouse, and assessments throughout her hospitalization, her diagnoses included unspecified depressive disorder, gambling disorder, and opioid use disorder. Although no longer listed in the DSM-5 as a diagnosis, it should be noted this patient suffers from polysubstance addiction, including a chemical addiction to opioids and a material addiction to gambling. It was agreed that her condition could be adequately managed in the outpatient setting, and the patient was discharged per request.

The patient followed up with her well-established behavioral healthcare team to manage her care and monitor her medication dosages. She restarted outpatient counseling services. A telephone call six months after discharge confirmed the patient had since been maintained on 80 mg of Prozac daily, 25 mg naltrexone for opioid use disorder, 200 mg modafinil PRN for shiftwork disorder, 2 mg lorazepam PRN for anxiety, and independent counseling sessions with her behavioral health specialist. There were no adverse outcomes and no new admissions within this time period.

## Discussion

Aripiprazole is typically used in combination with other psychotropic medications to enhance their effects on the brain [1]. The FDA Adverse Events Reporting System (FAERS) Public Dashboard indicates that there have been 2,193 total reported cases involving gambling disorder or gambling while using aripiprazole from the years 2005 to 2023 [3]. In 2016, the FDA put out a safety announcement stating “The U.S. Food and Drug Administration (FDA) is warning that compulsive or uncontrollable urges to gamble, binge eat, shop, and have sex have been reported with the use of the antipsychotic drug aripiprazole (Abilify, Abilify Maintena, Aristada, and generics)” [2]. In addition, Grall-Bronnec et al. also reported that patients who were young, impulsive, high novelty seekers, began gambling in adolescence, and with a history of substance misuse, just as the patient in our case, were more severe pathological gamblers when using aripiprazole compared to older patients on a full dopamine replacement therapy who began gambling later in their life [7]. However, they indicated the development of pathologic gambling is often multi-faceted with various additional environmental causes. Most cases of compulsive gambling in concordance with aripiprazole occur between a few days and up to a year after starting the medication [5]. The impulsivity profile, past medical history, and timeline match that of our patient.

While the patient's suicide attempt could have been related to the patient’s unspecified depressive disorder, it does not explain the patient's impulsive gambling. Additionally, the patient, as well as collateral information from her husband, reported she had been diagnosed with depression since about the same time she began casually gambling in childhood and had been able to control both conditions until about a year after beginning the new medication, aripiprazole. This presents the likeliness that her condition was exacerbated with the addition of aripiprazole. In a similar manner, an article from 2023 describes a patient who had been gambling casually their whole life, and their gambling turned impulsive after beginning aripiprazole [8]. 

In our patient, we observed an improvement in symptoms upon tapering aripiprazole, as well as sustained maintenance of gambling disorder six months past the hospital discharge. These results are consistent with the case series conducted by Gaboriau et al., where patients saw improvements in their gambling disorder days to months after the treatment was stopped, and some within just one dosage decrease [6]. It could be argued the patient’s improvement in symptoms could have been related to the intensive inpatient treatment with their medications monitored and phone inaccessible; however, the patient has noted no adverse events since stopping the medication and their discharge from the hospital. Six months post-discharge, they have not had any readmissions to an inpatient psychiatric unit, and have not taken aripiprazole since. This further strengthens the possibility that aripiprazole use increased the patient's gambling severity to a level of a diagnosable gambling disorder.

It should be noted that at the time of the admission, the patient was also prescribed modafinil, a drug sparsely associated with compulsive gambling disorder. Zack and Poulos described modafinil use as bidirectional; it increases gambling urges in those with low impulsivity and decreases gambling urges in those with high impulsivity [9]. With our patient’s attempted suicide due to gambling losses, and history of opioid use disorder, we conclude she is in the high impulsivity category. Further supporting the work of Zack and Poulos, there are a few case reports presenting modafinil as the cause of compulsive gambling disorder in low-impulsivity patients [10,11]. Considering our patient was taking modafinil for the past five years and is within the high impulsivity category, it is less likely modafinil is the cause of the patient developing gambling disorder. Furthermore, a 2020 study based on the work of Zack and Poulos used rat models and found consistent effects of modafinil reducing gambling choices, regardless of impulse category. This includes improved impulse control and decreased loss-chasing behavior, which is what our patient was engaging in at the time of her gambling and attempted suicide [12]. Although the study was performed on rats, it is novel and could provide further insight into why our patient was able to gamble recreationally for the past five years while taking modafinil and did not experience an increase in gambling severity until after beginning aripiprazole. The interaction of modafinil and gambling disorder in humans remains complicated and there is a need for more research on its exact effects.

The case presented was limited by the restrictions of clinical practice, the ability to monitor the patient’s adherence to their medication changes, and reliance on their perspective of how they are currently doing. However, despite these limitations, the symptoms the patient experienced and the clinical timeline of those symptoms align with those experienced by others using aripiprazole. Because of the patient's confounding factors, it remains difficult to say the use of aripiprazole was completely causal to this patient's compulsive gambling. Nonetheless, the patient developing gambling disorder with the use of aripiprazole, in combination with their predisposing factors, is consistent with previous research and further supports the notion that aripiprazole can negatively impact impulse control in an already vulnerable patient. The patient's improvement of symptoms following the termination of the drug supports this as well.

## Conclusions

Decreased impulse control resulting in gambling disorder is a well-documented potential side effect of aripiprazole. The risk seems to intensify in patients with previously documented impulse control risk factors, namely opioid use disorder or prior engagement in gambling rituals. Knowing there is a rare chance of excessive gambling and decreased ability to suppress urges when taking aripiprazole, the prescribing provider and patient need to have thorough conversations about previous compulsive behaviors, substance misuse, or gambling practices before deciding to begin aripiprazole. To ensure patient safety, this case should affect the practice of physicians and advanced practice providers who prescribe aripiprazole and impact the shared decision-making process between patients and their healthcare providers.
